# Secure Polar Coding for the Primitive Relay Wiretap Channel

**DOI:** 10.3390/e23040442

**Published:** 2021-04-09

**Authors:** Manos Athanasakos, George Karagiannidis

**Affiliations:** 1 Department of Informatics and Telecommunications, National and Kapodistrian University of Athens, 157 72 Athens, Greece; 2 Department of Electrical and Computer Engineering, Aristotle University of Thessaloniki, 541 24 Thessaloniki, Greece; geokarag@auth.gr

**Keywords:** polar codes, relay channel, information-theoretic security, decode-and-forward, strong secrecy

## Abstract

With the emergence of wireless networks, cooperation for secrecy is recognized as an attractive way to establish secure communications. Departing from cryptographic techniques, secrecy can be provided by exploiting the wireless channel characteristics; that is, some error-correcting codes besides reliability have been shown to achieve information-theoretic security. In this paper, we propose a polar-coding-based technique for the primitive relay wiretap channel and show that this technique is suitable to provide information-theoretic security. Specifically, we integrate at the relay an additional functionality, which allows it to smartly decide whether it will cooperate or not based on the decoding detector result. In the case of cooperation, the relay operates in a decode-and-forward mode and assists the communication by transmitting a complementary message to the destination in order to correctly decode the initial source’s message. Otherwise, the communication is completed with direct transmission from source to the destination. Finally, we first prove that the proposed encoding scheme achieves weak secrecy, then, in order to overcome the obstacle of misaligned bits, we implement a double-chaining construction, which achieves strong secrecy.

## 1. Introduction

The wiretap channel, introduced by A. Wyner in his seminal work [1], paved the way for the exploitation of the channel medium characteristics in terms of information-theoretic security. This approach has the major advantage that security does not rely on any shared secret key, i.e., keyless security. In view of the emergence of wireless communication and massive connectivity, this benefit has led to a rich literature, which investigates several channel models towards the design of low-complexity coding schemes for both reliability and secrecy.

After van der Meulen’s introduction of the relay channel in [2] and the extension of the work of Cover and El Gamal in [3], cooperative diversity is considered as an important advancement in wireless networks, since it can achieve higher rates in comparison to direct transmission. In practice, a network may be comprised with illegitimate users; cooperation between trusted users has been exploited as a way to establish secure communication. The rate-equivocation region was characterized in [4,5] for a four-terminal relay channel and an eavesdropper under several cooperation protocols. Half-duplex relay channel models are considered in [6], where the authors studied coding techniques for the relay channel with orthogonal components (primitive relay channel). The secrecy capacity for this class of channels was investigated in [7] for the binary-input discrete memoryless channel (B-DMC) and the Gaussian case. Although the importance of cooperation for reliability and security in large networks is well established, the aforementioned works presented bounds on the secrecy capacity while relying on random coding arguments. Undoubtedly, designing codes for these type of channels is of great importance as the evolution of networks require security solutions with low consumption and complexity.

Since the pioneering work of Arikan on the polar codes [8], which are capacity-achieving for the symmetric B-DMC, several polar coding schemes have been proposed to fulfill the secrecy requirement. These codes are constructed based on the phenomenon of channel polarization, that is, the channel is split into *N* “bit-channels”, which tend to be either error-free or fully noisy channels as *N* grows. This result is the basic tool in designing a polar coding scheme, which satisfies both reliability and secrecy conditions. In [9], a scheme for the degraded wiretap channel that meets the requirement for weak secrecy was proposed; in [10], the authors used a different partition of the index set to develop a scheme for strong secrecy. Under this framework, several coding schemes for multiuser channels have been investigated in [11,12,13]. However, although in the open literature there are some applications of polar codes without security constraints for the relay channel [14,15,16,17,18], the investigation of whether polar codes are suitable for the relay wiretap scenario has drawn little attention. Finally, the authors in [19] proposed a coding scheme capable of achieving weak secrecy for the relay-eavesdropper channel, whereas in [20], the proposed polar coding scheme guarantees strong secrecy for the case of symmetric channels and under the assumption that the eavesdropper’s channel is degraded. Note that the natural nested structure of polar codes and their low encoding/decoding complexity identify them as a promising choice for the practical implementation of merging coding and security into one scheme.

### 1.1. Related Work and Contributions

Since the introduction of the chaining technique [21] for polar codes, several explicit and efficient coding schemes for different information-theoretic models have been proposed. The work of [10] introduced the polar coding chaining construction in order to provide strong secrecy for the symmetric and degraded point-to-point wiretap channel. Later on, a polar code technique for asymmetric models was proposed in [22] and, along with the chaining construction, were the basic tools used to prove the secrecy capacity achievability of general wiretap channels. Specifically, in [12], the authors considered the chaining technique to deal with the nondegraded wiretap channel by artificially constructing the subset property and polar coding for asymmetric models to prove that polar codes achieve secrecy capacity in general. However, they considered only the weak secrecy case. Concurrently, refs. [11,13] developed a polar coding scheme for the general broadcast wiretap channel relying on different approaches. While both works considered the strong secrecy criterion, the first drew parallels between the achievability proof through output statistic of random binning and their proposed encoding scheme and avoided using randomized decisions during the encoding and decoding procedures ([23], Theorem 3), while the latter relied on shared random mappings ([22], Theorem 3) that may require exponential storage complexity. Recently, the authors of [24] extended the scheme of [13] by considering a more general model, in which the transmitter sends common and confidential messages over a broadcast wiretap channel. In their scheme, a new chaining construction is proposed to deal with the common information transmission.

Motivated by the above, in this paper, we consider the primitive relay wiretap channel and propose polar coding schemes based on different coordinate partitions. We prove that these schemes satisfy weak and strong secrecy requirements, while simultaneously guarantee a low probability of error at the legitimate receiver. Specifically, by careful partitioning of the coordinates, we first improve the analysis of [19] for the case of weak secrecy in a single transmission block. Then, we propose a new encoding algorithm under the strong secrecy criterion. In particular, we consider the general primitive relay wiretap channel, without assuming degradedness or symmetric channels as in [20]. The scheme utilizes previous polar coding techniques using the minimum rate of shared randomness and relies on both choosing the coordinate partitions properly and a transmission protocol that divides the communication into multiple blocks. Due to the nature of the channel under consideration, a new double-chaining construction is designed in order to satisfy the stronger secrecy requirement. Finally, an additional functionality is considered at the relay node; using the results from [15], the relay possesses a detector deciding if the result is erroneous or not and, based on that, discards it or cooperates in decode-and-forward (DF) mode.

### 1.2. Structure

The rest of the paper is organized as follows. In Section 2, some preliminaries on polar codes and the basic concept of the proposed scheme are introduced. The system model and the constraints in designing the coding scheme are presented in Section 3, followed by the main results of this paper; the encoding schemes for weak and strong secrecy and the analysis of reliability and security are presented in Section 4. Finally, Section 5 concludes the paper.

## 2. Polar Codes and the Relay Channel

### 2.1. Some Fundamentals on Polar Coding

We consider a binary-input channel with input alphabet X, output alphabet Y, and the conditional probability distribution $W_{X|Y}\left(\cdot |\cdot \right)$, with capacity $C\left(W\right)=\max_{P_{X}}I\left(X;Y\right)$. The symmetric capacity I(W) is the value of mutual information I(X;Y) when *X* is uniformly distributed. Moreover, if *W* is symmetric, then I(W)=C(W).

The Bhattacharyya parameter of the channel *W* is defined as

(1) $$Z\left(W\right)=\sum_{y\in Y}\sqrt{W_{X|Y}\left(0|y\right)W_{Y|X}\left(y|1\right)}.$$

For length $N=2^{n}$ with n∈N, let $G_{N}=B_{N}F^{⊗n}$ be the polarizing matrix, where $B_{N}$ is the bit-reversal mapping, $F=\left[\begin{array}{cc} 1 & 0\\ 1 & 1 \end{array}\right]$ and $F^{⊗n}$ denote the *n*th Kronecker power of *F*. By applying transformation $G_{N}$ to *N* bits $u_{1}^{N}$ and sending it through *N* independent uses of a B-DMC W:X→Y, thus, an *N*-dimensional channel is created, defined by

(2) $$W_{N}^{\left(i\right)}\left(y_{1}^{N},u_{1}^{i-1}|u_{i}\right)=\frac{1}{2^{N-1}}\sum_{u_{i+1}^{N}\in {\left\{0,1\right\}}^{N-i}}W_{N}\left(y_{1}^{N}|u_{1}^{N}\right),$$

where $W_{N}^{\left(i\right)}$ denotes the *i*-th bit-channel created by synthesizing *N* uses of the channel *W*. As *N* grows, $W_{N}^{\left(i\right)}$ approaches either an error-free or a completely noisy channel. The idea is to transmit information only over the “good” channels while keeping the inputs of “bad” channels fixed and known to all parties. Thus, the *N* bit-channels are partitioned into “good” channels $G_{N}\left(W\right)$ and “bad” channels $B_{N}\left(W\right)$ based on the value of their Bhattacharyya parameter.

(3) $$\begin{array}{cc} G_{N}\left(W\right) & =\{i\in \left[N\right]:Z\left(W_{N}^{\left(i\right)}\right)\leq \delta_{N}\}\\ B_{N}\left(W\right) & =\{i\in \left[N\right]:Z\left(W_{N}^{\left(i\right)}\right)\geq 1-\delta_{N}\}, \end{array}$$

where [N]={1,2,…,N}, $\delta_{N}=2^{-N^{\beta }}$ with 0<β<1/2 and $Z(W_{N}^{\left(i\right)})$ is the Bhattacharyya parameter of channel $W_{N}^{\left(i\right)}$. It has been shown [25,26] that for any symmetric binary-input channel *W* and for any β<1/2,

(4) $$\begin{array}{cc} \lim_{N\to \infty }=\frac{G_{N}\left(W\right)}{N} & =C(W)\\ \lim_{N\to \infty }=\frac{B_{N}\left(W\right)}{N} & =1-C(W). \end{array}$$

Based on the above, we can transmit a message of $k=|G_{N}\left(W\right)|$ bits, which is written in the bits $u_{i}$, $i\in G_{N}\left(W\right)$ and the rest N−k bits of $u_{1}^{N}$ are frozen and set to 0. Thus, a code word $x_{1}^{N}=u_{1}^{N}G_{N}$ is sent over the channel. On the other side, the received sequence $y_{1}^{N}$ can be decoded by finding an estimate of $u_{1}^{N}$ by computing the values ${\hat{u}}_{i}$, i∈[N] based on the following successive cancellation (SC) rule:

(5) $${\hat{u}}_{i}=\left\{\begin{array}{cc} 0, & \mathrm{if}\frac{W_{N}^{\left(i\right)}\left(y_{1}^{N},{\hat{u}}_{1}^{i-1}|0\right)}{W_{N}^{\left(i\right)}\left(y_{1}^{N},{\hat{u}}_{1}^{i-1}|1\right)}\geq 1\mathrm{and}i\in G_{N}\left(W\right)\\ 0, & \mathrm{if}i\in B_{N}\left(W\right)\\ 1, & \mathrm{otherwise}. \end{array}\right.$$

Using this decoding rule [8,25], we can upper bound the error probability

(6) $$P_{e}\leq \sum_{i\in G_{N}\left(W\right)}Z\left(W_{N}^{\left(i\right)}\right)\leq \delta_{N},$$

where β∈(0,1/2).

### 2.2. Bounds and Nested Structure

In this paper, one of the main characteristics of the proposed coding scheme is the nested structure of the polar codes. Next, we briefly explain this structure and its usage for binning in multiterminal communication scenarios. In particular, the authors in [14] designed a coding scheme for the three-terminal stochastically degraded relay channel with orthogonal receivers. First however, let us review the capacity bounds of the relay channel.

It is well-known that a bound for the capacity of the general relay channel is given by the cut-set upper bound [3]

(7) $$C\leq \max_{P_{X},P_{X_{R}}}\min\left\{I\left(X;Y_{SR}Y_{SD}\right),I\left(X;Y_{SD}\right)+I\left(X_{R};Y_{RD}\right)\right\},$$

and for the primitive relay channel, the DF lower bound reduces to [6]

(8) $$R^{DF}=\max_{P_{X}}\min\left\{I\left(X;Y_{SR}\right),I\left(X;Y_{SD}\right)+I\left(X_{R};Y_{RD}\right)\right\}.$$

Next, we briefly describe the nested structure of polar codes proposed in [14] for DF relaying, which, for any rate $R<R^{DF}$, there exists a sequence of polar codes with a vanishing probability of error at the destination. The following Lemma from [27] is used to exploit the nested nature of polar codes.

**Lemma** **1.** *Let Q and V be BSM channels such that Q is degraded with respect to V. Further, let $Q_{1},\ldots Q_{i}$ and $V_{1},\ldots V_{i}$ denote the N corresponding bit-channels. Then, $Q_{i}$ is degraded with respect to $V_{i}$, that is, $I\left(Q_{i}\right)\leq I\left(V_{i}\right)$ and $Z\left(Q_{i}\right)\geq Z\left(V_{i}\right)$.*

**Figure 1:**
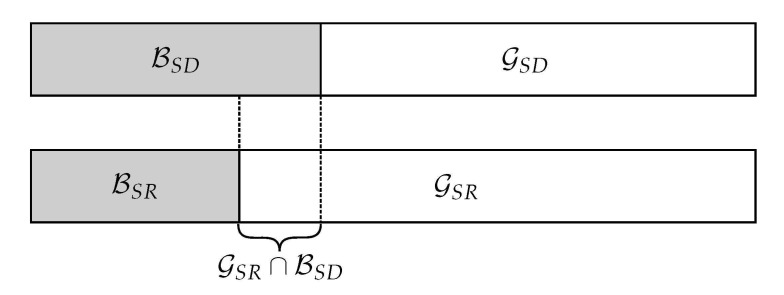
Nested structure of polar codes for the relay channel.

From the above lemma, it follows directly that if the channel *Q* is degraded with respect to *V*, the set of good channels for *Q* is a subset of the set of good channels of *V*, i.e., for all constants β, we have $G_{N}\left(Q\right)\subseteq G_{N}\left(V\right)$. Degradation implies that a channel is better than another one. The primitive relay channel is said to be degraded when the source-destination link is worse than that between source and relay. The encoding process starts at the source when a rate $R<I(W_{SR})$ is chosen and a capacity-achieving polar code for the channel $W_{SR}$ is used. We define $G_{SR}$ and $B_{SR}$ as in (3) for channel $W_{SR}$ as the information and frozen set, respectively. Let M contain the information and the frozen bits transmitted by the source. Moreover, the bits $m_{i}$, $i\in G_{SR}$ carry the message and $m_{i}$, $i\in B_{SR}$ are the frozen bits, which are known to the relay and the destination prior to transmission. As shown in Figure 1, the destination cannot decode this sequence in its entirety due to its degraded channel. So, we also select a set of indices for direct communication over $W_{SD}$ and define $G_{SD}$ and $B_{SD}$ similarly. From Lemma 1, it holds that $G_{SD}\subseteq G_{SR}$, as $W_{SD}$ is degraded compared to $W_{SR}$, i.e., the decoder at the destination knows the values of the symbols $m_{i}$, $i\in G_{SD}\cup G_{SR}$ and in order to employ SC decoding, the relay must forward the information $m_{i}$, $i\in G_{SR}\cup B_{SD}$.

### 2.3. Smart Relaying

It has been shown in [15] that by providing the relay with a simple error detector, we can significantly improve the error performance by letting the decoder at the relay select whether it will cooperate or not. We consider a DF cooperative transmission, where the message is encoded by using the nested polar coding scheme of Section 2.2. The relay carries a detector for erroneous decoding, i.e., if the likelihood ratio is less than a threshold, then the relay discards the decoded result and does not transmit to the destination, otherwise, the communication takes place under the assistance of the relay node.

We consider a threshold *s* for the relay decoder and the log-likelihood ratio (LLR) as defined for the successive decoding process [8]:

(9) $$L_{N}^{\left(i\right)}\left(y_{1}^{N},{\hat{u}}_{1}^{i-1}\right)=\log\frac{W_{N}^{\left(i\right)}\left(y_{1}^{N},{\hat{u}}_{1}^{i-1}|0\right)}{W_{N}^{\left(i\right)}\left(y_{1}^{N},{\hat{u}}_{1}^{i-1}|1\right)},$$

where the decoder decides based on the rule in (5). A flag *F* is used to determine if the decoding result will be discarded or not according to

(10) $$F=\left\{\begin{array}{cc} 0, & \mathrm{if}-s\leq L_{N}^{\left(i\right)}\left(y_{1}^{N},{\hat{u}}_{1}^{i-1}\right)\leq s\\ 1, & \mathrm{otherwise}. \end{array}\right.$$

The above procedure does not introduce any additional complexity during the relay’s decoding, thus, this functionality improves the error probability of the overall communication for free. In the next section, we bind together the smart relaying and the exploitation of the nested structure of polar codes to design a scheme that satisfies secrecy and reliability requirements for the primitive relay channel in the presence of an eavesdropper.

## 3. System Model and Requirements

In this section, we describe the primitive relay wiretap channel and set the goals of our encoding scheme; reliability and secrecy. Then, we present the architecture of the proposed communication model with the smart error detector at the relay.

### 3.1. The Relay Wiretap Channel

The relay wiretap channel models a multihop transmission scheme, where a relay cooperates with the source to communicate with the destination in the presence of an eavesdropper. We consider a four-terminal B-DMC with orthogonal receiver components, with the transition probability mass function

(11) $$p\left(y,y_{sr},z|x_{s},x_{r}\right)=p\left(y_{sd},y_{sr},z_{se}|x_{s}\right)p\left(y_{rd},z_{re}|x_{r}\right).$$

**Figure 2:**
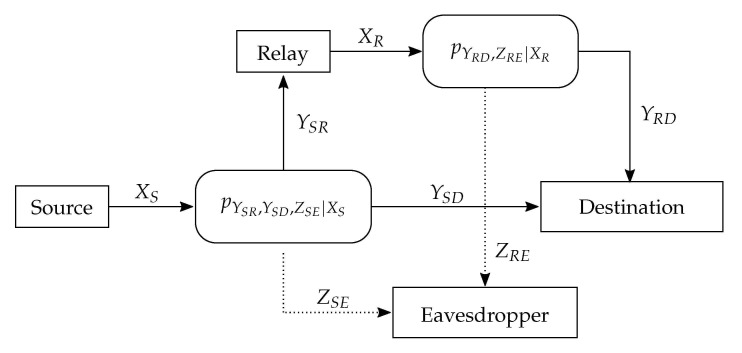
The relay wiretap channel model with orthogonal components.

In this model, $X_{S}$ and $X_{R}$ are the channel inputs from the source and relay, respectively, while Y, $Y_{SR}$, and Z are the channel outputs at the destination, relay, and eavesdropper, respectively. The observation vectors at the destination’s and eavesdropper’s output are $Y=(Y_{SD},Y_{RD})$ and $Z=(Z_{SE},Z_{RE})$, respectively. Figure 2 illustrates the channel, which consists of a source, a relay, the legitimate receiver, and the eavesdropper. The source wishes to reliably communicate a message M with the legitimate receiver under the assistance of a trusted relay while keeping it safe from the eavesdropper. The transmission takes place in two stages as follows:

- In the first stage, the Source encodes a message M into a code word $X_{S}$ and broadcasts it to the destination and relay.
- In the second stage, the Relay first decodes the $Y_{SR}$ and obtains ${\hat{M}}_{R}$, then re-encodes it and transmits $X_{R}$ to the destination.
- The Destination combines the two observations to produce an estimate $\hat{M}$ of the original message.
- The Eavesdropper observes $Z=(Z_{SE},Z_{RE})$ during both transmissions.

### 3.2. Coding Requirements

We aim to design a coding scheme that satisfies both reliability and secrecy requirements. Probability of error is used to quantify the *reliability* of the scheme, where the goal is to satisfy

(12) $$\lim_{N\to \infty }Pr\left\{M\neq \hat{M}\right\}=0.$$

To measure the statistical independence between the message transmitted and eavesdropper observation, we use the following metrics:

(13) $$\begin{array}{cc} \lim_{N\to \infty }\frac{I(M;Z)}{N} & =0, \end{array}$$

(14) $$\begin{array}{cc} \lim_{N\to \infty }I\left(M;Z\right) & =0. \end{array}$$

In (13), security is measured in terms of the normalized mutual information between the transmitted message M and received vector by the eavesdropper Z. The encoding scheme is designed to satisfy this requirement in order to operate with *weak secrecy*. However, as shown by Maurer in [28], it is too weak for cryptographic applications as it is possible for the eavesdropper to retrieve a considerable amount of information even if (13) is satisfied. As a solution, we can use a stronger metric, that is, the encoding scheme operates with *strong secrecy* if (14) is satisfied.

### 3.3. Architecture

**Figure 3:**
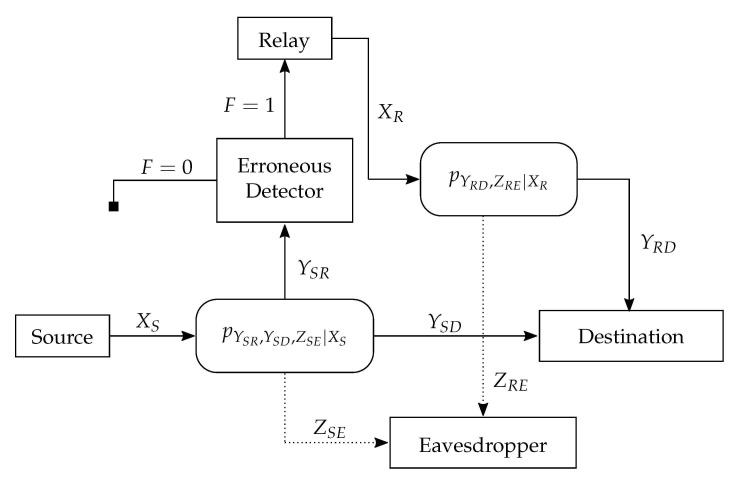
The relay wiretap channel with the erroneous detector.

Our model is very similar to that described in Section 3.1, where in order to enjoy the benefits of smart relaying, we add an error detector at the relay. This allows the relay to select whether it is beneficial to cooperate with the source or not. As illustrated in Figure 3, the relay operates based on the detector result, i.e., if F=0, the relay discards the decoding result and the communication is completed via direct transmission from source to the destination. In this case, the secure polar coding scheme of [9] or [10] for the classic wiretap channel can be used. On the other hand, if F=1, the communication is assisted by the DF relay and the designing of such a coding scheme for reliability and secrecy is proposed in this paper.

## 4. Polar Coding for Secrecy

In this section, we present the encoding scheme, which simultaneously satisfies the reliability and weak secrecy constraints for the model of Section 3.2. Then, we identify the difficulties to achieve strong secrecy and introduce a new construction that satisfies this condition by using a double-chaining technique.

### 4.1. Weak Secrecy

As already mentioned above, polarization results into noiseless and pure-noisy bit-channels. Having in mind that the system needs to meet only the reliability requirement, one shall fill the good channels with information bits and keep the value of the bad channels fixed. However, when a third unauthorized party eavesdrops the communication, the secrecy constraint must be satisfied. The idea behind coding for secrecy is to confuse the nonlegitimate user with random messages, so their observation is different from the real message. Utilizing polar codes, we can design a secure coding scheme by properly partitioning the bit-channels. First, in order to achieve reliability, we send the message over the good channels (low entropy) for the legitimate users and bad channels (high entropy) for the eavesdropper. To confuse the eavesdropper and secure the transmission, we choose to hide the message by sending random bits over the reliable channels. That is, the goal is to construct an encoding process that makes the communication reliable and secure simultaneously, i.e., satisfying conditions (12) and (13). For the relay wiretap channel and under the DF protocol, these conditions must be satisfied in both transmission stages.

First, as in (3), we define the following subset of indices:

(15) $$\begin{array}{cc} G_{N}\left(W_{kl}\right) & =\{i\in \left[N\right]:Z\left(W_{N}^{\left(i\right)}\right)\leq \delta_{N}\}\\ B_{N}\left(W_{kl}\right) & =\{i\in \left[N\right]:Z\left(W_{N}^{\left(i\right)}\right)\geq 1-\delta_{N}\}, \end{array}$$

where k∈{S,R} and l∈{R,D,E}, for k≠l. Next, we partition the set [N] based on [9] as follows:

(16) $$\begin{array}{cc} I_{1} & =G_{N}\left(W_{SR}\right)\cap B_{N}\left(W_{SE}\right)\\ F_{1} & =B_{N}\left(W_{SR}\right)\\ R_{1} & =G_{N}\left(W_{SE}\right). \end{array}$$

**Figure 4:**
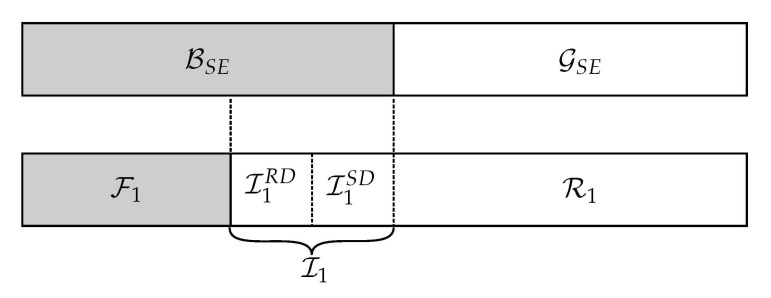
Stage I: Index partitioning (16) at the source.

**Encoding at source**: We choose a rate $R<I(W_{SR})$ and use the indices in $G_{N}\left(W_{SR}\right)$ to encode the information and broadcast it to the relay and the destination. All parties know the values of frozen bits $F_{1}$. We fill with random bits the indices in $R_{1}$ in order to protect the message. The information is stored in the bits of set $I_{1}$. However, due to Lemma 1, degradation implies that the destination cannot decode the whole information due to $G_{N}\left(W_{SD}\right)\subseteq G_{N}\left(W_{SR}\right)$, we distribute the message in $I_{1}^{SD}=G_{N}\left(W_{SD}\right)\cap B_{N}\left(W_{SE}\right)$ and $I_{1}^{RD}=G_{N}\left(W_{SR}\right)\cap B_{N}\left(W_{SD}\right)$, with $I_{1}=I_{1}^{SD}\cup I_{1}^{RD}$ (Figure 4). The message bits in $I_{1}^{RD}$ must be provided by the relay during the second transmission using the following partition.

(17) $$\begin{array}{cc} I_{2} & =G_{N}\left(W_{RD}\right)\cap B_{N}\left(W_{RE}\right)\\ F_{2} & =B_{N}\left(W_{RD}\right)\\ R_{2} & =G_{N}\left(W_{RE}\right). \end{array}$$

**Figure 5:**
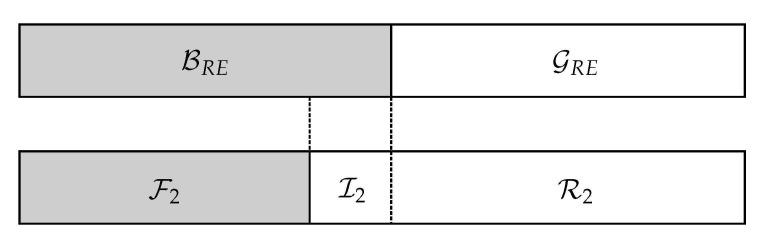
Stage II: Index partitioning (17) at the relay.

**Processing at the relay**: The relay using SC and the knowledge of frozen bits decodes the message transmitted by the source, then extracts and encodes the information bits with indices in $I_{1}^{RD}$ and forwards them to the destination using a capacity-achieving polar code for $W_{RD}$ and partition (17). To protect this transmission, we again fill with random bits the indices in $R_{2}$, the information bits are in the set $I_{2}$ and the bits in $F_{2}$ are frozen and known (Figure 5).

**Decoding at the destination**: Having received $Y_{RD}$ and recovered the missing bits from the relay, the destination uses these bits in addition to the first observation $Y_{SD}$ and employs the SC algorithm to recover the source’s message.

Based on the above encoding and decoding process, we prove that the coding scheme satisfies both requirements (12) and (13).

#### 4.1.1. Reliability Analysis

The reliability follows immediately from the design of the coding scheme and the results developed in [8]. Specifically, the low error probability claim must be satisfied for the relay and destination. First, since $I_{1}\cup R_{1}=G_{N}\left(W_{SR}\right)$, the error probability at the relay is upper bounded as

(18) $$P_{e}^{SR}\leq \sum_{i\in I_{1}\cup R_{1}}Z\left(W_{SR}^{\left(i\right)}\right)\leq 2^{-N^{\beta }}.$$

The probability of error for the relay–destination transmission of the second time-slot, since $I_{2}\cup R_{2}=G_{N}\left(W_{RD}\right)$ by design, is upper bounded as

(19) $$P_{e}^{RD}\leq \sum_{i\in I_{2}\cup R_{2}}Z\left(W_{RD}^{\left(i\right)}\right)\leq 2^{-N^{\beta }}.$$

The bits from the relay are then decoded by the destination and, together with the source’s transmission $Y_{SD}$, the original message is retrieved using the SC algorithm. Consequently, the overall error probability at the destination is upper bounded by

(20) $$P_{e}\leq O\left(2^{-N^{\beta }}\right).$$

Moreover, we obtain the constraints on the transmission rate, i.e., $R<I(W_{SR})$ and $R<I\left(W_{SD}\right)+I\left(W_{RD}\right)$, which yields the symmetric DF rate for relay channels $R^{DF}$.

#### 4.1.2. Secrecy Analysis

Let us turn to the security analysis. Let U denote the intermediate vector constructed by the encoding process, with $U_{I}=U_{I_{1}\cup I_{2}}$, $U_{R}=U_{R_{1}\cup R_{2}}$, and $U_{F}=U_{F_{1}\cup F_{2}}=0$. Moreover, the source’s message $M=(M_{1},M_{2})$ and takes values in ${\left\{0,1\right\}}^{\left|I\right|}$, with $M_{1}$ and $M_{2}$ denoting messages from the source and relay, respectively. We need to prove that the normalized mutual information, (13), between M and Z vanishes asymptotically. We evaluate the statistical independence between the message and eavesdropper’s observations using random frozen vector over all possible choices of frozen bits as follows:

(21) $$\begin{array}{cc} I(M;Z|U_{F}) & =I(U_{I};Z|U_{F}) \end{array}$$

(22) $$\begin{array}{cc} \; & =I\left(U_{I},U_{R};Z|U_{F}\right)-I\left(U_{R};Z|U_{F},U_{I}\right) \end{array}$$

(23) $$\begin{array}{cc} \; & =I\left(U;Z\right)-I\left(U_{R};Z|U_{F},U_{I}\right) \end{array}$$

(24) $$\begin{array}{cc} \; & =I\left(U;Z\right)-H\left(U_{R}|U_{F},U_{I}\right)+H\left(U_{R}|Z,U_{F},U_{I}\right) \end{array}$$

(25) $$\begin{array}{cc} \; & =I\left(U;Z\right)-H\left(U_{R}\right)+H\left(U_{R}|Z,U_{F},U_{I}\right) \end{array}$$

(26) $$\begin{array}{cc} \; & =I\left(U;Z\right)-\left|R\right|+H\left(U_{R}|Z,U_{F},U_{I}\right) \end{array}$$

(27) $$\begin{array}{cc} \; & \leq N\left(I\left(W_{SE}\right)+I\left(W_{RE}\right)\right)-\left|R\right|+H\left(U_{R}|Z,U_{F},U_{I}\right), \end{array}$$

where (22) is derived from the chain rule of mutual information and (23) is due to $I\left(U_{I},U_{R};Z|U_{F}\right)=I\left(U_{I},U_{R};Z|U_{F}\right)+I\left(U_{F};Z\right)=I\left(U_{I},U_{R},U_{F};Z\right)=I\left(U;Z\right)$ and that $I(U_{F};Z)=0$. Consequently, (25) follows from the independence of $U_{R},U_{F}$, and $U_{I}$, while (27) is concluded by the fact that $I(W_{SE})$ and $I(W_{RE})$ are the capacity of $W_{SE}$ and $W_{RE}$, respectively, and the data processing inequality.

Examining (27), we observe that in order to upper bound the mutual information of (21), we need to find an upper bound for the entropy term $H(U_{R}|Z,U_{F},U_{I})$. Hence, we have the following lemma:

**Lemma** **2.** *The conditional entropy is upper bounded as*

$$H\left(U_{R}|Z,U_{F},U_{I}\right)\leq h_{2}\left(2^{-N^{\beta }}\right)+\left|R\right|2^{-N^{\beta }},$$

*where $h_{2}\left(\cdot \right)$ is the binary entropy function and |R| is the size of random vector $U_{R}$.*

**Proof.**  Let us assume that the eavesdropper has knowledge of $U_{I}$ in addition to Z and the frozen bits. Therefore, the eavesdropper can compute an estimate of ${\hat{U}}_{R}$ (since R is transmitted via the good channels $G_{N}\left(W_{SE}\right)$ and $G_{N}\left(W_{RE}\right)$), by using the SC algorithm with

(28) $$\begin{array}{c} P_{e}^{Eve}=P\left[{\hat{U}}_{R}\neq U_{R}\right]\leq \sum_{i\in R}\left(Z\left(W_{SE}^{\left(i\right)}\right)+Z\left(W_{RE}^{\left(i\right)}\right)\right)\leq 2^{-N^{\beta }}. \end{array}$$

We then introduce a random variable *E* for the error as follows:

(29) $$\begin{array}{c} E=\left\{\begin{array}{cc} 1, & \mathrm{if}\;{\hat{U}}_{R}\neq U_{R}\\ 0, & \mathrm{if}\;{\hat{U}}_{R}=U_{R}, \end{array}\right. \end{array}$$

and we derive the following

(30) $$\begin{array}{cc} H(E,U_{R}|Z,U_{F},U_{I}) & =H\left(U_{R}|Z,U_{F},U_{I}\right)+H\left(E|U_{R},Z,U_{F},U_{I}\right) \end{array}$$

(31) $$\begin{array}{cc} \; & =H(U_{R}|Z,U_{F},U_{I}), \end{array}$$

since the second entropy term in (30) equals zero. Moreover, note that

(32) $$\begin{array}{cc} H(E,U_{R}|Z,U_{F},U_{I}) & =H\left(U_{R}|E,Z,U_{F},U_{I}\right)+H\left(E|Z,U_{F},U_{I}\right) \end{array}$$

(33) $$\begin{array}{cc} \; & \leq H\left(U_{R}|E,Z,U_{F},U_{I}\right)+H\left(E\right), \end{array}$$

since the second entropy term in (32) can be upper bounded by $H\left(E\right)=H\left(P_{e}^{Eve}\right)$. Thus, from (31) and (33), we get

(34) $$\begin{array}{cc} H(U_{R}|Z,U_{F},U_{I}) & \leq H\left(U_{R}|E,Z,U_{F},U_{I}\right)+H\left(E\right)\\ \; & =P\left[E=0\right]H\left(U_{R}|E=0,Z,U_{F},U_{I}\right) \end{array}$$

(35) $$\begin{array}{c} +P\left[E=1\right]H\left(U_{R}|E=1,Z,U_{F},U_{I}\right)+H\left(P_{e}^{Eve}\right) \end{array}$$

(36) $$\begin{array}{cc} \; & \leq P_{e}^{Eve}\left|R\right|+H\left(P_{e}^{Eve}\right), \end{array}$$

where (36) is because $H(U_{R}|E=0,Z,U_{F},U_{I})=0$ and $H\left(U_{R}|E=1,Z,U_{F},U_{I}\right)\leq H\left(U_{R}\right)=\left|R\right|$. Rearranging (36) and using (28), we get the desired upper bound and the proof is completed. □

Finally, considering Lemma 2 and (27), we get the following upper bound:

(37) $$\begin{array}{cc} I(M;Z|U_{F}) & \leq N\epsilon_{N}+h_{2}\left(2^{-N^{\beta }}\right)+\left|R\right|2^{-N^{\beta }}, \end{array}$$

where $\epsilon_{N}=I\left(W_{SE}\right)+I\left(W_{RE}\right)-\left|R\right|/N$ and if we divide both sides of (37) by *N*, all terms tend to zero as N→∞, since $\lim_{N\to \infty }\epsilon_{N}=0$, with $R_{1}\in G_{N}\left(W_{SE}\right)$ and $R_{2}\in G_{N}\left(W_{RE}\right)$.

Thus, the proposed polar coding scheme satisfies the weak secrecy requirement for all possible choices of frozen vector,

(38) $$\lim_{N\to \infty }\frac{I(M;Z)}{N}=0$$

and the achievable rate under this encoding procedure is given by

(39) $$R_{s}^{weak}=R^{DF}-\left(I\left(W_{SE}\right)+I\left(W_{RE}\right)\right),$$

for large enough *N*.

### 4.2. Strong Secrecy

The above scheme can only achieve the weak secrecy requirement, due to the assumptions that $B_{N}^{c}\left(W_{SE}\right)\subset G_{N}\left(W_{SR}\right)$ and $B_{N}^{c}\left(W_{RE}\right)\subset G_{N}\left(W_{RD}\right)$, while in general, this is not true. Although the number of coordinates in $G_{N}^{c}\left(W_{SR}\right)\cap B_{N}^{c}\left(W_{SE}\right)$ and $G_{N}^{c}\left(W_{RD}\right)\cap B_{N}^{c}\left(W_{RE}\right)$ is very small, this constitutes the difficulty in obtaining reliability and strong secrecy simultaneously. The authors in [10] proposed a different partition of the coordinates which resolves the above problem. For the case of the symmetric relay wiretap channel and degraded eavesdropper link, the strong secrecy claim was proved in [20]. In the following, we provide a solution for a more general case, where we do not make any assumption on eavesdropper’s channel quality and consider a nonsymmetric channel model.

#### 4.2.1. Asymmetric Channel Coding

In [22], the authors presented a polar coding scheme, which achieves the capacity of a B-DMC, to cover the general case of arbitrary input distributions. Let $U^{N}=X^{N}G_{N}$ and define the following sets:

(40) $$\begin{array}{cc} H_{X} & =\{i\in \left[N\right]:Z\left(U_{i}|U^{i-1}\right)\geq 1-\delta_{N}\}\\ L_{X} & =\{i\in \left[N\right]:Z\left(U_{i}|U^{i-1}\right)\leq \delta_{N}\}\\ H_{X|Y} & =\{i\in \left[N\right]:Z\left(U_{i}|U^{i-1},Y^{N}\right)\geq 1-\delta_{N}\}\\ L_{X|Y} & =\{i\in \left[N\right]:Z\left(U_{i}|U^{i-1},Y^{N}\right)\leq \delta_{N}\}, \end{array}$$

where Z(X|Y) is the Bhattacharyya parameter of a random variable pair (X,Y), defined as

(41) $$\begin{array}{c} Z\left(X|Y\right)=2\sum_{y\in Y}P_{Y}\left(y\right)\sqrt{P_{X|Y}\left(0|y\right)P_{X|Y}\left(1|y\right)}. \end{array}$$

From [29], we have

(42) $$\begin{array}{cc} \lim_{N\to \infty }\frac{H_{X}}{N} & =H(X), \end{array}$$

(43) $$\begin{array}{cc} \lim_{N\to \infty }\frac{H_{X|Y}}{N} & =H(X|Y). \end{array}$$

For a nonsymmetric B-DMC channel W:X→Y, it is not possible to use all the good bit-channels to transmit information. Hence, in [22], a different partition of the set [N] was proposed:

(44) $$\begin{array}{cc} I & =H_{X}\cap L_{X|Y}\\ F_{r} & =H_{X}\cap L_{X|Y}^{c}\\ F_{d} & =H_{X}^{c}. \end{array}$$

For $i\in H_{X}$, $U_{i}$ is almost uniformly distributed and independent of the past $U^{i-1}$, therefore, it can carry information. For $i\in L_{X|Y}$, $U_{i}$ is almost determined by $U^{i-1}$ and $Y^{N}$, implying that it can be decoded in a successive manner, and from (42) and (43), we have

(45) $$\lim_{N\to \infty }\frac{\left|I\right|}{N}=I\left(X;Y\right).$$

The remaining indices are frozen; for $i\in F_{r}$, $U_{i}$ is almost uniformly distributed and independent of the past $U^{i-1}$ but cannot be reliably decoded given $Y^{N}$; for $i\in F_{d}$, $U_{i}$ is almost determined by $U^{i-1}$. As suggested in [22], the values of bits in $\left\{i\in F_{r}\cup F_{d}\right\}$ are assigned by random mappings $\lambda_{i}:{\left\{0,1\right\}}^{i-1}\to \left\{0,1\right\}$ according to the following probability rule:

(46) $$\begin{array}{c} \lambda_{i}\left(u^{i-1}\right)=u,\;w.p.\;P_{U_{i}|U^{i-1}}, \end{array}$$

which are shared between the encoder and the decoder. However, this operation requires sharing a large amount of randomness which is often undesirable. As a remedy to this, simplified schemes that require a vanishing rate of shared randomness were proposed in [23,30].

Let the bits in {i∈I} be used to store information as mentioned above, and let bits in $\left\{i\in F_{r}\right\}$ be uniformly distributed random bits shared between the encoder and the decoder. This sequence can be reused over several blocks, making the rate loss negligible. The values of $\left\{i\in F_{d}\right\}$ are sampled from the distribution $P_{U_{i}|U^{i-1}}$, and the bits in $\left\{i\in H_{X}^{c}\cap L_{X|Y}^{c}\right\}$ are transmitted to the receiver separately with some reliable code, with negligible rate loss (since $|L_{X|Y}^{c}\setminus H_{X|Y}|=o\left(n\right)$ and $H_{X|Y}\subseteq H_{X}$, we have that $|H_{X}^{c}\cap L_{X|Y}^{c}|=o\left(n\right)$).

After the transmission of $x^{N}=u^{N}G_{N}$, the receiver knows the sequences $\left\{i\in F_{r}\right\}$ and $\left\{i\in H_{X}^{c}\cap L_{X|Y}^{c}\right\}$ and successively constructs the estimate $\hat{u}$ using the following rule for each bit-channel:

(47) $${\hat{u}}_{i}=\left\{\begin{array}{cc} \arg\max_{u\in [0,1]}P_{U_{i}|U^{i-1},Y^{N}}\left(u|{\hat{u}}^{i-1},y^{N}\right), & \mathrm{if}i\in L_{X|Y}\\ u_{i}, & \mathrm{if}i\in L_{X|Y}^{c}. \end{array}\right.$$

This scheme’s rate approaches I(X;Y) and the error probability can be upper bounded by $P_{e}\leq \sum_{i\in L_{X|Y}}Z\left(U_{i}|U^{i-1},Y^{N}\right)=O\left(\delta_{N}\right)$.

#### 4.2.2. Encoding Scheme

In the following, we develop the main contribution of this work, the encoding scheme for the primitive relay wiretap channel, and remove the assumptions on degradedness and symmetry. The transmission takes place over k+1 blocks of *N* bits. Prior to the communication, trusted parties share a secret seed of random bits D, which is used as a “chain” between transmitted blocks. In particular, encoding is performed so that the bits of D are passed on the legitimate receiver (relay or destination) using their reliable and secure indices. The chaining is implemented by sending the bits in D(j) of block *j* as part of the message block j−1 for all j∈[1,…,k]. This construction allows the legitimate receiver to employ SC for block *j* and recover these bits reliably, while security is guaranteed.

Let us apply the aforementioned construction to the relay wiretap channel under investigation. We consider the following partition of the index set

(48) $$\begin{array}{cc} I_{1} & =H_{X_{S}}\cap G_{N}\left(W_{SR}\right)\cap B_{N}\left(W_{SE}\right)\\ F_{1} & =H_{X_{S}}\cap G_{N}^{c}\left(W_{SR}\right)\cap B_{N}\left(W_{SE}\right)\\ R_{1} & =H_{X_{S}}\cap G_{N}\left(W_{SR}\right)\cap B_{N}^{c}\left(W_{SE}\right)\\ D_{1} & =H_{X_{S}}\cap G_{N}^{c}\left(W_{SR}\right)\cap B_{N}^{c}\left(W_{SE}\right)\\ B_{1} & =H_{X_{S}}^{c}, \end{array}$$

where in the set $I_{1}$, information bits are stored; set $F_{1}$ is the set of frozen bits; $R_{1}$ are the randomly chosen bits; $D_{1}$ are the misaligned bits; and $B_{1}$ are the almost deterministic bits. We note that, as in the weak secrecy case in Section 4.1, the information bits in $I_{1}$ are distributed in both $I_{1}^{SD}$, which can be decoded by the destination, and $I_{1}^{RD}$, which is the message that the relay forwards through the $W_{RD}$. Thus, for this transmission, the relay uses the following partition:

(49) $$\begin{array}{cc} I_{2} & =H_{X_{R}}\cap G_{N}\left(W_{RD}\right)\cap B_{N}\left(W_{RE}\right)\\ F_{2} & =H_{X_{R}}\cap G_{N}^{c}\left(W_{RD}\right)\cap B_{N}\left(W_{RE}\right)\\ R_{2} & =H_{X_{R}}\cap G_{N}\left(W_{RD}\right)\cap B_{N}^{c}\left(W_{RE}\right)\\ D_{2} & =H_{X_{R}}\cap G_{N}^{c}\left(W_{RD}\right)\cap B_{N}^{c}\left(W_{RE}\right)\\ B_{2} & =H_{X_{R}}^{c}. \end{array}$$

Before describing the encoding procedure, we define the following set $D=D_{1}\cup D_{2}$, which is used as the secret seed and is shared among the source, relay, and destination. Additionally, we fix two arbitrary sets $E_{1}\subset I_{1}$ and $E_{2}\subset I_{2}$ with $|E_{1}\left|=\right|D_{1}|$ and $|E_{2}\left|=\right|D_{2}|$, and $E=E_{1}\cup E_{2}$ with |E|=|D|. Consequently, the messages of the two-hop transmission are indexed by the bits in ${\tilde{I}}_{1}=I_{1}\setminus E$ and ${\tilde{I}}_{2}=I_{2}\setminus E$, respectively. As a consequence of removing the eavesdropper’s degraded channel assumption, the cardinality of $D_{1}$ and $D_{2}$ is not o(N) anymore and we must ensure that by removing the bits $E_{1}$ and $E_{2}$ from $I_{1}$ and $I_{2}$, respectively, there is no loss in the rate. However, the preshared rate can be made very small by choosing a large enough *k*, i.e., |D|/kN.

**Figure 6:**
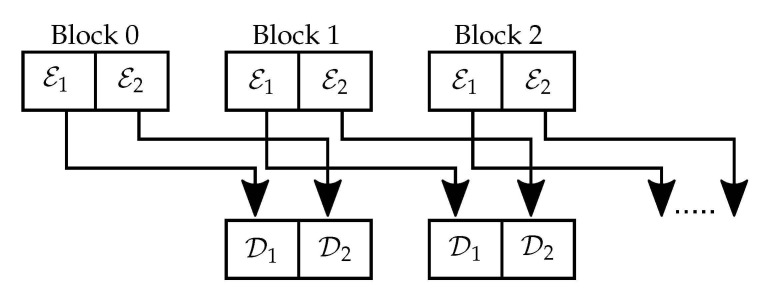
The double-chaining construction.

Overall, the transmission is performed in two stages, where in order to satisfy the strong secrecy requirement while the probability of error vanishes, we manipulate the misaligned bits in both transmissions by creating a double-chaining structure, i.e., the bits in D and their links E of the previous block create a chain for each transmission, as in Figure 6.

Let us describe this double-chaining construction, assuming that the legitimate parties have knowledge of the seed D(1). By transmitting E(0) with a separate code, the first chain is formed by $D_{1}\left(j\right)=E_{1}\left(j-1\right)$ during the source transmission towards the relay and the destination, and the second chain is formed by $D_{2}\left(j\right)=E_{2}\left(j-1\right)$ when the relay sends the missing bits to the legitimate receiver. After each source block transmission, the first $|D_{1}|$ bits of D are used to create the chain and are being replaced block by block. Similarly, the second-hop chain is created after each block is transmitted by the relay by using the remaining $|D_{2}|$ bits of D.

**Figure 7:**
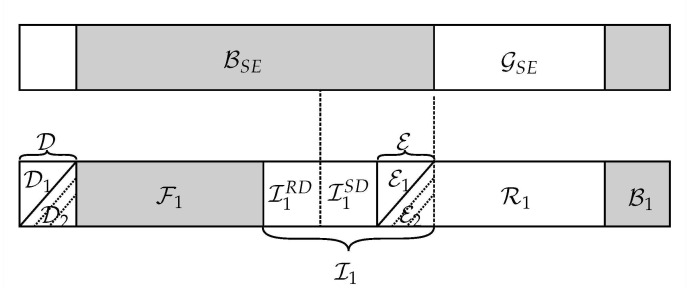
Index partitioning (48) at the source.

**Source encoding:** For block j=1,…,k, set ${\tilde{I}}_{1}$ carries the message bits; set $R_{1}$ is filled with uniformly distributed random bits; the first $|D_{1}|$ bits of the set D are chained with the bits of $E_{1}$, i.e., $D_{1}\left(j\right)=E_{1}\left(j-1\right)$; the bits in $F_{1}$ are fixed, known, and can be reused over blocks; and the bits of $B_{1}$ are sampled from $P_{U_{i,S}|U_{S}^{i-1}}$. Moreover, since the source–destination link is weaker, the bits in $G\left(W_{SR}\right)\cap B\left(W_{SD}\right)$ need to be delivered to the destination by the relay during the second-hop transmission. That is, the message bits of ${\tilde{I}}_{1}$ are loaded in ${\tilde{I}}_{1}^{SD}=G\left(W_{SD}\right)\cap B\left(W_{SE}\right)$ and ${\tilde{I}}_{1}^{RD}=G\left(W_{SR}\right)\cap B\left(W_{SD}\right)$. Finally, as described in Section 4.2.1, let $\Phi_{1}$ be the vector storing the not completely polarized bit-channels $\left\{i\in H_{X_{S}}^{c}\cap G_{N}^{c}\left(W_{SR}\right)\right\}$, which is shared secretly between the legitimate users with some reliable error-correcting code. Figure 7 shows the coding scheme, the lines on $D_{2}$ and $E_{2}$ imply the first chain construction.

**Figure 8:**
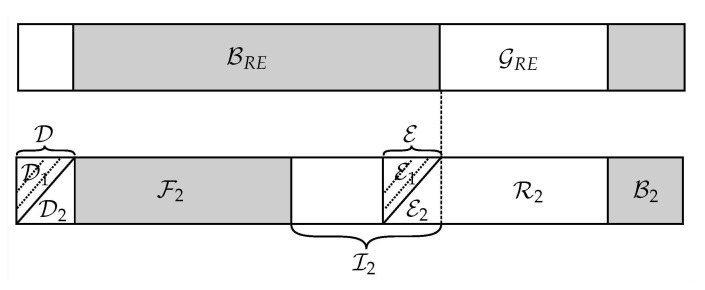
Index partitioning (49) at the relay.

**Processing at the relay:** The relay decodes message block *j*, knowing $F_{1}$, the seed $D_{1}\left(j\right)=E_{1}\left(j-1\right)$, and the bits of $\Phi_{1}$, then extracts the bits in ${\tilde{I}}_{1}^{RD}$ and forwards them to the destination by using a polar code for the channel $W_{RD}$ using partition (49). Specifically, for block j=1,…,k message bits are loaded in the set ${\tilde{I}}_{2}$; random bits in the set $R_{2}$; the bits in the set $D_{2}$ are chained with those of $E_{2}$, i.e., $D_{2}\left(j\right)=E_{2}\left(j-1\right)$, as shown in Figure 8; and the bits of $B_{2}$ are sampled from $P_{U_{i,R}|U_{R}^{i-1}}$. Furthermore, let $\Phi_{2}$ be the vector storing the not completely polarized bit-channels $\left\{i\in H_{X_{R}}^{c}\cap G_{N}^{c}\left(W_{RD}\right)\right\}$, which is shared secretly with the destination using some reliable error-correcting code. The frozen set for this transmission is $F_{2}$, which is known to the destination.

**Destination decoding:** At the destination, the process starts by decoding the first block message of the relay transmission, knowing $F_{2}$ and $D_{2}\left(j\right)=E_{2}\left(j-1\right)$ and the bits of $\Phi_{2}$. Then, it uses those bits and the knowledge of $F_{1}$, $D_{1}$, and $\Phi_{1}$ to decode the corresponding message block received from the source transmission at the first stage by employing the SC algorithm.

**Remark** **1.** *The encoding scheme above requires a certain amount of shared randomness between the legitimate users. Specifically, $F_{1}$, $F_{2}$ are available to all users (including the eavesdropper), while $D_{1}$, $D_{2}$, and $\Phi_{1}$, $\Phi_{2}$ are known only to the legitimate users. Note that $F_{i}$, for i=1,2, can be reused over blocks and since $|F_{1}\left|+\right|F_{2}|=O\left(N\right)$, the rate needed can become arbitrarily small by choosing a large k. The rate of secret seed $D_{i}$, and the rate of the not completely polarized bit-channels $\Phi_{i}$, for i=1,2, can also become negligible by choosing a sufficiently large k. In general, the rate loss caused by the shared randomness is minor compared to the overall message rate.*

Let us now introduce the following random variables needed for the reliability and secrecy analysis. For the transmission in blocks j=1,…,k, denote the source’s message bits in ${\tilde{I}}_{1}$ by $M_{1,k}$ and let $M_{2,k}$ be the message transmitted by the relay with bits in ${\tilde{I}}_{2}$, frozen bits in $F_{1}$ and $F_{2}$ are denoted by $F_{1,k}$, and let $F_{2,k}$. Further, let $E_{1,k}$ and $E_{2,k}$ correspond to the bits belonging to $E_{1}\left(j\right)$ and $E_{2}\left(j\right)$, respectively, for j=0,…,k. To make the analysis compact, we also denote $M_{k}=\left(M_{1,k},M_{2,k}\right)$, $F_{k}=\left(F_{1,k},F_{2,k}\right)$, $E_{k}=\left(E_{1,k},E_{2,k}\right)$; the *k*-length vectors $M_{1}^{k}=\left(M_{1},\ldots ,M_{k}\right)$, $F_{1}^{k}=\left(F_{1},\ldots ,F_{k}\right)$, $E_{0}^{k}=\left(E_{0},\ldots ,E_{k}\right)$; and let $Z_{0}^{k}=\left(Z_{0},\ldots ,Z_{k}\right)$ be the sequence of eavesdropper’s observations $Z=(Z_{SE},Z_{RE})$ during the *k*-th block transmission from source and relay.

#### 4.2.3. Total Variation Distance and Reliability Analysis

We now analyze the proposed scheme by first examining the closeness in terms of total variation distance of the distribution induced by the encoding process and the target distribution. We would like to find an upper bound on the variation distance between these two distributions. Let V(P,Q) be the total variation distance and D(P||Q) be the Kullback–Leibler divergence between distributions *P* and *Q*. Following the analysis of [13], we have that

(50) $$\begin{array}{cc} D(P_{X_{S}}||Q_{X_{S}}) & =D(P_{U_{S}}||Q_{U_{S}}) \end{array}$$

(51) $$\begin{array}{cc} \; & =\sum_{i=1}^{N}D\left(P_{U_{i,S}\left|\right|U_{S}^{i-1}}|Q_{U_{i,S}|U_{S}^{i-1}}\right) \end{array}$$

(52) $$\begin{array}{cc} \; & =\sum_{i\in H_{X_{S}}}\left(1-H\left(U_{i,S}|U_{S}^{i-1}\right)\right) \end{array}$$

(53) $$\begin{array}{cc} \; & \leq \sum_{i\in H_{X_{S}}}\left(1-Z^{2}\left(U_{i,S}|U_{S}^{i-1}\right)\right) \end{array}$$

(54) $$\begin{array}{cc} \; & \leq 2|H_{X_{S}}|\delta_{N}\leq 2N\delta_{N}, \end{array}$$

where (50) is due to the polar transform $X_{S}=U_{S}G_{N}$, (51) holds by the chain rule of KL divergence, (52) holds since the values of $H_{X_{S}}$ are chosen uniformly, (53) follows from the inequality $Z{\left(X|Y\right)}^{2}\leq H\left(X|Y\right)$ ([29], Proposition 2), and (54) follows by the design of $H_{X_{S}}$. Similarly, we have

(55) $$D(P_{X_{R}}\left|\right|Q_{X_{R}})\leq 2N\delta_{N}.$$

To obtain the desired bound in terms of total variation distance between the two joint distributions, we note that

(56) $$\begin{array}{cc} V(P_{X_{S},X_{R},Y_{SR},Y_{SD},Y_{SE},Y_{RD},Y_{RE}},Q_{X_{S},X_{R},Y_{SR},Y_{SD},Y_{SE},Y_{RD},Y_{RE}}) & =V(P_{X_{S},X_{R}},Q_{X_{S},X_{R}}) \end{array}$$

(57) $$\begin{array}{cc} \; & \leq 4\sqrt{\ln2}\sqrt{N\delta_{N}}\overset{\Delta }{=}\delta_{N}^{\left(1\right)}, \end{array}$$

where (56) and (57) follow from [31] (Lemma 17) and Pinsker’s inequality, using (54) and (55), respectively. This result indicates that the induced joint distribution is asymptotically indistinguishable from the target one.

We next examine the reliability of this scheme by estimating the error probability for the legitimate parties. First, for the relay, since the rate of the transmission uses a polar coding sequence with $R<I(W_{SR})$, and assuming that $Pr\{{\hat{E}}_{0}\neq E_{0}\}\to 0$—i.e., there is a code with $\epsilon_{N}\to 0$ used to convey the seed to the legitimate users—the probability of erroneous decoding at the relay in the k+1 blocks is

(58) $$P_{e}^{SR}\leq \epsilon_{N}+kO\left(2^{-N^{\beta }}\right),$$

for all β<1/2.

Similarly, the destination will recover the relay’s message $M_{2}$, with the error probability bounded by

(59) $$P_{e}^{RD}\leq kO\left(2^{-N^{\beta }}\right),$$

since ${\tilde{I}}_{2}\cup R_{2}\subset G\left(W_{RD}\right)$, and knowing $F_{2}$ and $D_{2}\left(j\right)$. Then, using those bits, it can decode the source’s message $M_{1}$ using the SC algorithm. Overall, the probability of error at the destination after the second transmission is then bounded as

(60) $$P_{e}\leq \epsilon_{N}+kO\left(2^{-N^{\beta }}\right),$$

where $\epsilon_{N}$ is the vanishing error of the code transmitting the seed prior to the communication.

#### 4.2.4. Secrecy Analysis

We will show that the strong secrecy requirement is satisfied by utilizing the double-chaining construction described above. For the proposed encoding scheme, the information leakage to the eavesdropper can be analyzed as follows:

(61) $$\begin{array}{cc} I(M_{1}^{k};Z_{0}^{k}|F_{1}^{k}) & \leq I(M_{1}^{k},E_{k};Z_{0}^{k}|F_{1}^{k})\\ \; & =I\left(M_{1}^{k},E_{k};Z_{k}|F_{1}^{k}\right)+I\left(M_{1}^{k},E_{k};Z_{0}^{k-1}|Z_{k},F_{1}^{k}\right) \end{array}$$

(62) $$\begin{array}{cc} \; & =I\left(M_{k},E_{k};Z_{k}|F_{1}^{k}\right)+I\left(M_{1}^{k},E_{k};Z_{0}^{k-1}|Z_{k},F_{1}^{k}\right)\\ \; & \leq I\left(M_{k},E_{k};Z_{k}|F_{1}^{k}\right)+I\left(M_{1}^{k},E_{k}Z_{k};Z_{0}^{k-1}|F_{1}^{k}\right)\\ \; & \leq I\left(M_{k},E_{k};Z_{k}|F_{1}^{k}\right)+I\left(M_{1}^{k},E_{k-1},E_{k},Z_{k};Z_{0}^{k-1}|F_{1}^{k}\right) \end{array}$$

(63) $$\begin{array}{cc} \; & =I\left(M_{k},E_{k};Z_{k}|F_{1}^{k}\right)+I\left(M_{1}^{k-1},E_{k-1};Z_{0}^{k-1}|F_{1}^{k}\right) \end{array}$$

(64) $$\begin{array}{cc} \; & =I\left(M_{k},E_{k};Z_{k}|F_{k}\right)+I\left(M_{1}^{k-1},E_{k-1};Z_{0}^{k-1}|F_{1}^{k-1}\right), \end{array}$$

where (62) and (63) are due to the Markov chains $M_{1}^{k-1}\to M_{k}E_{k}\to Z_{k}$ and $M_{k}E_{k}Z_{k}\to M_{1}^{k-1}E_{k-1}\to Z_{0}^{k-1}$, respectively. (64) is obtained by noticing that using the chain rule for mutual information, we get

(65) $$\begin{array}{cc} I(M_{k},E_{k};Z_{k}|F_{1}^{k}) & =I\left(M_{k},E_{k};Z_{k}|F_{k}\right)+I\left(M_{k},E_{k};F_{1}^{k-1}|Z_{k},F_{k}\right)-I\left(M_{k},E_{k};F_{1}^{k-1}|F_{k}\right) \end{array}$$

(66) $$\begin{array}{cc} \; & =I(M_{k},E_{k};Z_{k}|F_{k}), \end{array}$$

where the last two terms equal zero, similar to the second term of (64). Next, from (61), we have the following:

(67) $$I\left(M_{1}^{k};Z_{0}^{k}|F_{1}^{k}\right)\leq \sum_{j=1}^{k}I\left(M_{j}E_{j};Z_{j}|F_{1}^{k}\right)+\underset{\epsilon_{N}}{\underset{︸}{I(E_{0};Z_{0})}},$$

where the last term is the secret seed shared between the legitimate parties prior to the communication and we assume that there exists a secure coding scheme with $\epsilon_{N}\to 0$. In order to complete the proof of strong secrecy, it remains to be shown that the first term of the RHS in (67) vanishes as well. Therefore, we need to bound the capacity of the eavesdropper’s channel induced by our encoding. For this purpose, we prove the following lemma.

**Lemma** **3.** *For any j=1,…,k, we have*

$$I\left(M_{j},E_{j};Z_{j}|F_{j}\right)\leq \delta_{N}^{\left(3\right)},$$

*where $\delta_{N}^{\left(3\right)}\overset{\Delta }{=}2N\delta_{N}+\delta_{N}^{\left(1\right)}\left(N-\log\delta_{N}^{\left(1\right)}\right)$.*

**Proof.**  Let $I=|I_{1}\left|+\right|I_{2}|$ with indices labeled as $\left\{a_{1},\ldots ,a_{I}\right\}$ with $a_{1}<...<a_{I}$; similarly, let $F=|F_{1}\left|+\right|F_{2}|$ and label the indices as $\left\{b_{1},\ldots ,b_{F}\right\}$ with $b_{1}<...<b_{F}$. Moreover, let S=I+F with labels $\left\{c_{1},\ldots ,c_{S}\right\}$ and assume that $c_{1}<...<c_{S}$. -4.6cm0cm

(68) $$\begin{array}{cc} I(M_{j},E_{j};Z_{j}|F_{j}) & =H\left(M_{j},E_{j}|F_{j}\right)-H\left(M_{j},E_{j}|Z_{j},F_{j}\right)\\ \; & =H\left(M_{j},E_{j}\right)-H\left(M_{j},E_{j},F_{j}|Z_{j}\right)+H\left(F_{j}|Z_{j}\right)\\ \; & =\sum_{i=1}^{I}H\left(T_{a_{i}}|T^{a_{1}},\ldots ,T_{a_{i-1}}\right)-\sum_{i=1}^{S}H\left(T_{c_{i}}|Z_{j},T_{c_{1}},\ldots ,T_{c_{i-1}}\right)+\sum_{i=1}^{F}H\left(T_{b_{i}}|Z_{j},T_{b_{1}},\ldots ,T_{b_{i-1}}\right)\\ \; & \leq \sum_{i=1}^{S}\left(1-H\left(T_{c_{i}}|Z_{j},T^{c_{i-1}}\right)\right), \end{array}$$

where (68) is due to the fact that conditioning reduces entropy. Considering the entropy term in (68) and noticing that it is derived under the induced distribution of the coding scheme, we would like to find the distance between the induced entropy and the entropy under the target distribution $\tilde{H}\left(T_{c_{i}}|Z_{j},T^{c_{i-1}}\right)$, where $\tilde{H}$ denotes the entropy under the target distribution $P_{X^{N},Z^{N}}$. First, using the inequality $Z{\left(X|Y\right)}^{2}\leq H\left(X|Y\right)$ ([29], Proposition 2), we get that

(69) $$\begin{array}{cc} \tilde{H}\left(T_{c_{i}}|Z_{j},T^{c_{i-1}}\right) & \geq Z{\left(T_{c_{i}}|Z_{j},T^{c_{i-1}}\right)}^{2}\\ \; & \geq 1-2\delta_{N}. \end{array}$$

To estimate the distance, we rely on Th. 17.3.3 [32], and we have

(70) $$\begin{array}{cc} |H\left(T_{c_{i}}|Z_{j},T^{c_{i-1}}\right)-\tilde{H}\left(T_{c_{i}}|Z_{j},T^{c_{i-1}}\right)| & \leq V\left(P_{X^{N},Z^{N}},Q_{X^{N},Z^{N}}\right)\times \log\frac{2^{N}}{V(P_{X^{N},Z^{N}},Q_{X^{N},Z^{N}})} \end{array}$$

(71) $$\begin{array}{cc} \; & \leq \delta_{N}^{\left(1\right)}\left(N-\log\delta_{N}^{\left(1\right)}\right)\overset{\Delta }{=}\delta_{N}^{\left(2\right)}, \end{array}$$

where (71) follows from (57) and the fact that f(x)=x(N−logx) is increasing for 0<x<1.

Thus, from (68), (69), and (70), we conclude that

(72) $$I\left(M_{j},E_{j};Z_{j}|F_{j}\right)\leq 2N\delta_{N}+\delta_{N}^{\left(2\right)}\overset{\Delta }{=}\delta_{N}^{\left(3\right)}.$$

□

Finally, combining Lemma 3 and (67), we get the desired result:

(73) $$I\left(M_{1}^{k};Z_{0}^{k}|F_{1}^{k}\right)\leq I\left(M_{1}^{k}E_{k};Z_{0}^{k}|F_{1}^{k}\right)\leq \epsilon_{N}+k\delta_{N}^{\left(3\right)},$$

where, for *k* fixed and N→∞, we observe that $I(M_{1}^{k};Z_{0}^{k}|F_{1}^{k})$ vanishes as we have assumed that $\epsilon_{N}\to 0$, and that completes the secrecy analysis.

Moreover, the achievable rate under this encoding scheme is given by

(74) $$R_{s}^{strong}=\frac{k}{k+1}\left[R^{DF}-\left(I\left(W_{SE}\right)+I\left(W_{RE}\right)\right)-2\Delta \right],$$

as *N* grows large and by choosing *k* to be large enough, where 2Δ is a vanishing small rate penalty induced by the double-chaining structure and the shared seed. Undoubtedly, designing a coding scheme that takes into account reliability and secrecy as requirements, a trade-off between achievable rate and secrecy is introduced. In our scheme (Although we are discussing the strong secrecy encoding scheme, the same applies for the scheme of Section 4.1, where the rate loss is lower due to the absence of the double-chaining construction). the random bits and the bits allocated to convey the misaligned indices are the factors that mean the achievable secret rate is lower than the $R^{DF}$, which is the lower bound without secrecy constraints. To reduce this loss and attain a higher achievable rate, a line of work exists that proposes a cross-layer security scheme, which combines information-theoretic security with classical encryption mechanisms [33,34].

## 5. Conclusions

The construction of practical coding schemes for information-theoretic security is of great significance. In this work, we have proposed an efficient coding scheme based on polar codes for the primitive relay wiretap channel, which guarantees a level of information-theoretic security. In our setup, we exploited the nested structure of polar codes for cooperative relaying in a DF strategy. We presented an encoding scheme that achieves reliability and weak secrecy, but fails to provide strong secrecy. Through a different partition of the coordinates and a chaining construction, we were able to prove that reliability and strong secrecy can be obtained simultaneously for the channel model, without any assumption regarding symmetry and the eavesdropper’s channel condition. Moreover, in our strong secrecy scheme, we considered a simplified mechanism that only requires a vanishing rate of shared randomness, instead of sharing random mappings that may heavily increase the storage complexity. Finally, as en extra functionality, a “smart” relay is added, where an erroneous detector at the relay’s decoder operates to reduce the error probability while the complexity remains the same.
